# Clinical Applications of Procalcitonin in Pediatrics: An Advanced Biomarker for Inflammation and Infection—Can It Also Be Used in Trauma?

**DOI:** 10.1155/2014/286493

**Published:** 2014-10-28

**Authors:** Ioannis Koutroulis, Steven M. Loscalzo, Panagiotis Kratimenos, Sabina Singh, Evan Weiner, Vassiliki Syriopoulou, Stamatios Theocharis, Georgios Chrousos

**Affiliations:** ^1^Department of Pediatrics, St. Christopher's Hospital for Children, 3601 A Street, Philadelphia, PA 19134, USA; ^2^Drexel University College of Medicine, Philadelphia, PA, USA; ^3^Department of Neonatology, St. Christopher's Hospital for Children, Philadelphia, PA, USA; ^4^First Department of Pediatrics, University of Athens School of Medicine, Athens, Greece

## Abstract

*Background.* Procalcitonin is a small molecular peptide that has gained increased support as an adjunct diagnostic marker of infection in the adult population; the concordant body of evidence for the use of procalcitonin in pediatric populations is far less complete. *Objectives.* Our objective is to review the current evidence supporting the utilization of procalcitonin in children in a variety of clinical scenarios including SIRS, sepsis, burns, and trauma and to identify existing knowledge gaps. *Methods.* A thorough review of the literature was performed utilizing PubMed. We focused on using meta-analysis from adult populations to review current practices in interpretation and methodology and find concordant pediatric studies to determine if the same applications are validated in pediatric populations. *Results.* Current evidence supports the usage of procalcitonin as both a sensitive and a specific marker for the differentiation of systemic inflammatory response syndrome from sepsis in pediatrics with increased diagnostic accuracy compared to commonly used biomarkers including complete blood counts and C-reactive protein. *Conclusions.* Although the body of evidence is limited, initial observations suggest that procalcitonin can be used in pediatric trauma and burn patients as both a prognostic and a diagnostic marker, aiding in the identification of infection in patients with extensive underlying inflammation.

## 1. Introduction

Procalcitonin is a small molecular peptide that has gained increased support as an adjunct diagnostic marker of infection in the adult population; the concordant body of evidence for the use of procalcitonin in pediatric populations is far less complete.

Procalcitonin (PCT) is a 116-amino acid prohormone of calcitonin. Under normal conditions, the C-cells of the thyroid and K-cells of the lung are responsible for PCT gene expression. The prohormone is expressed in response to elevated serum calcium concentrations and undergoes an intracellular proteolytic cleave to the active calcitonin hormone. Calcitonin then acts to lower blood calcium levels through three separate mechanisms including inhibition of intestinal absorption, renal tubular cell reabsorption, and osteoclast activity [1].

In contrast to the biologically active form, PCT has no known receptor. During inflammation PCT is expressed in extrathyroid tissue and concentrations increase. The mechanism of PCT release outside of the thyroid has not been completely elicited but is presumed to be in response to a combination of proinflammatory markers including IL-1 and TNF-a and in response to cellular necrosis via mitochondrial DNA-linked danger associated molecular patterns (DAMPs) that are believed to behave in a similar fashion to the pattern recognition receptors (PRR) of pathogen associated molecular patterns (PAMPs) of the innate immune system [2, 3]. Increases in PCT levels have been well documented in response to the presence of bacterial sepsis and trauma and in a few additional scenarios including fungemia, surgery, birth stress, heat shock, and graft versus host disease. In response to proper stimulation, PCT concentrations rapidly increase 1000–10,000-fold over a period of 4–12 hours, reaching maximum levels at 24 hours. Combined with a relatively short half-life of 24–30 hours in circulation, this creates a favorable kinetics profile making PCT an ideal inflammatory biomarker [4].

In vivo, the full length PCT-116 protein can be truncated to des-Ala-Pro-PCT PCT3-116 which has a slightly different kinetic profile, peaking before the full length peptide. Current immunoassays do not differentiate between the two fractions, though isolation of specific PCT fractions may enhance the peptide's utility as a biomarker [5].

There are currently several companies manufacturing PCT assays, including Roche, Biomerieux, and Luminometric. One of the most sensitive assays, BRAHMS PCT KRYPTOR (Hennigsdorf, Germany), utilizes a two-stage fluorescent marker to detect PCT levels. The assay run time is approximately 20 with a reported functional assay sensitivity of 0.06 ug/L [6]. The same company also produces alternative assays for the measurement of PCT including an enzyme-linked fluorescent immunoassay (VIDAS systems) and an immunochromatographic semiquantitative point-of-care test (PCT-Q), each designed for use in different clinical scenarios.

## 2. Procalcitonin and Sepsis

The vast majority of research on PCT has focused on its use as biomarker for invasive infection in the setting of sepsis. A careful understanding of the utility of PCT in the setting of sepsis is required in order to explore possible alternative applications in the settings of traumatic injury or burns. The potential role of PCT as a biomarker for sepsis was first reported in the early 1990s. The appropriate utility of PCT as a marker depends on its ability to accurately aid in screening, diagnosis, risk stratification, and therapeutic monitoring [7]. Literature cited in this review supports the role of PCT as an adjunct clinical tool for determining the presence of an underlying infection in febrile pediatric patients and suggests that PCT may be a superior biomarker compared to C-reactive protein (CRP).

Sepsis is a complex syndrome resulting from the innate host response to invasive infection. The result is inadequate oxygen delivery to organs as well as defective utilization of oxygen by the tissues. Despite recent advances in care, sepsis remains a major cause of significant morbidity and mortality. Developmental differences between children and adults lead to distinct variations in the epidemiology, pathophysiology, and management of sepsis in children compared to adults [8]. The clinical signs of sepsis which include tachycardia, leukocytosis, tachypnea, and fever show marked variation in pediatric patients depending on the patient's age, potential source of infection, and any underlying comorbidities, making it difficult to rely exclusively on these indicators.

Early prospective studies identified that serum PCT concentrations increased at the onset of bacterial infection and that increases in PCT correlated with the severity of infection [9]. Prospective studies of pediatric ICU patients helped to quantify the positive correlation between disease severity and increasing PCT levels. A 2006 study of PICU patients reported median PCT plasma levels of 0.17, 0.43, 0.79, 1.80, 15.40, and 19.13 ng/mL in negative, systemic inflammatory response syndrome, localized infection, sepsis, severe sepsis, and septic shock groups, respectively [10]. The rapid elevation in PCT levels is observed across all pediatric ages, including neonates [11], though it should be noted that the diagnostic accuracy of PCT for neonatal sepsis appears to favor late-onset sepsis (>72 hrs) [12].

A recent retrospective study by Cies et al. in children demonstrated that a PCT value of ≥1 ng/mL predicted having a serious bacterial infection (OR = 1.18, 95% CI 1.07–1.49) and exhibited a PPV of 28%, a NPV of 93%, a sensitivity of 70%, and a specificity of 68% [13]. In a separate prospective cohort study, Rey et al. reported that a PCT level of >1.63 ng/mL had 85% sensitivity and 83% specificity for determining the presence of sepsis in pediatric ICU patients [10]. It is appropriate to note that there are modest variations in the reported diagnostic accuracy of PCT. These variations can be contributed to a variety of factors 7 including laboratory cut-offs, designated end points, patient population, and when PCT levels are drawn relative to the patient's clinical course.

When compared to other commonly used biomarkers, the literature suggests that PCT may provide a slight diagnostic advantage, due in part to its rapid kinetic profile. PCT reaches peak serum concentrations at 6–12 hours, compared to 24–48 hours for CRP. Because levels peak earlier, PCT can provide diagnostic information earlier during the course of infection, potentially before advanced clinical signs are apparent. While the precise diagnostic accuracy of PCT continues to be debated, compared to other biomarkers used in the diagnosis of sepsis including white blood cell counts, absolute neutrophil counts, and C-reactive protein (CRP), PCT is routinely reported as both more sensitive and specific [10, 11, 14].

Critically elevated PCT levels are nearly pathognomonic for sepsis and can be used to guide management. Though individual variation in cut-offs do exist, meta-analysis indicates that a selected value between 1 and 2 ng/mL has the highest diagnostic accuracy when it comes from differentiating sepsis from SIRS [15]. The variation in cut-offs suggests that PCT should not be utilized alone but rather considered as a supplement to help guide appropriate clinical judgment.

## 3. Procalcitonin and Pediatric Burns

Despite progressive improvements in burn medicine, pediatric burns continue to be associated with significant morbidity and mortality. In 2000 alone, across the United States an estimate 10,000 pediatric patients were admitted with accidental burn injuries, with a total cost of care estimated over $2 billion [16].

Thermal injuries are characterized by large variations in basic physiology both at the site of injury and systemically. Historically, the primary determinants of mortality have been age, the extent of burns, and the presence or absence of inhalation injury. Secondary to initial injury, patient recovery is complicated by fluid and electrolyte derangements, organ dysfunction, and a significant predisposition to infection [17]. Pneumonia and burn wound sepsis may account for more than 25% of burn-associated mortalities. The American Burn Association has thoroughly addressed the concern and developed alternate criteria for the diagnosis of sepsis in burn patients. The ABA has also endorsed continued studies on various inflammatory markers such as PCT to aid in diagnosis [17].

Burns, regardless of the total body surface area burned (TBSA), induce significant elevations in the traditional biomarkers of inflammation, including CRP, TNF-a, IL-6, and PCT, even without the presence of infection. Because of this underlying inflammation, the ability of PCT to differentiate sepsis from noninfectious SIRS is not overly apparent. An extensive systematic review of adult burn patients in 2009 by Mann et al. identified 3 separate reviews that concluded PCT assay had moderate ability to differentiate sepsis from noninfectious SIRS in burn patients. Aggregate conclusions, however, were limited by significant variations in the study populations and definitions of sepsis used in the study parameters [18].

Because of the significant individual variations in PCT levels secondary to the underlying inflammation associated with thermal injury, the utility of PCT as an adjunct tool for the diagnosis of sepsis during hospitalization is difficult to assess. An extensive review in 2011 examined available literature and found a limited body of evidence that supports the utility of PCT as an adjunct diagnostic tool for sepsis [18].

A 2007 study by Abdel-Hafez et al. trended PCT levels in pediatric burn patients and found significant elevations in PCT levels 48 hours after admission, regardless of an infectious process, average PCT levels of 69.1 ± 11.4 ng/mL. These values are significantly higher than what would be expected for aseptic patients and were found not to correlate with TBSA [19].

These results are in concordance with a 2004 pediatric burn study by Neely et al., who reported that PCT levels elevated rapidly following the initial burn and remained elevated for the first 72 hours of hospitalization. During this period, researchers were* unable* to rely on PCT alone to predict the onset of sepsis [20]. Their findings suggest that the underlying chronic inflammation from the burn generated significant elevations in PCT levels that served to mask predicted increases associated with the onset of sepsis. In this particular paper the standard for diagnosis of sepsis was determined subjectively by the burn surgeon at the center, which may limit the validity of their findings. It does, however, emphasize how the current lack of defined laboratory standards in burn patients is limiting continued research in the area.

While elevated PCT levels may not automatically imply sepsis, there is a large body of evidence that supports rising PCT levels are associated with worse outcomes. An upward trend in PCT levels from admission, regardless of the exact value, is an independent risk factor for both overall mortality and the development of sepsis in burn patients [21].

Currently, the following conclusions can be drawn based on the available evidence with respect to usage of procalcitonin in burn patients.

- Procalcitonin levels are modestly elevated in burn patients, even in those without infection, consistent with a massive systemic inflammatory response to thermal injury.
- Elevated procalcitonin levels correlate with high rates of mortality and infection but at this point do not reliably predict infection at the time of admission.
- There is a lack of data concerning the use of procalcitonin in pediatric burn patients, an area that should be expanded on in the future.

## 4. Procalcitonin and Pediatric Trauma

The acute triage of injuries is an essential part of trauma care and there are several well-established scoring systems that are routinely used to predict outcome following trauma to predict prognosis and mortality. There are not, however, strong models for detecting the development or progression to sepsis in trauma patients. While recent advances in surgical procedures and resuscitation techniques continue to improve patient outcomes, the development and application of biomarkers as both predictive and prognostic indicators remain an area of extensive research. Procalcitonin has been studied in a variety of different traumatic injuries. The majority of the current literature is focused on adults, with very little, if any, conclusive evidence in the pediatric populations.

Understanding the utility of PCT in the setting of trauma requires recognition that traumatic injury represents a significant inflammatory process with alterations of normal physiology. Because of the underlying inflammation associated with traumatic injury, the diagnosis of sepsis becomes particularly challenging, as many patients meet criteria for SIRS* independent* of an infectious insult.

Observational data shows PCT elevations above baseline occur with injuries to the head, face, thorax, abdomen, and extremities, with the highest elevations in blunt abdominal trauma. In isolated trauma, without evidence of infection, the initial elevations in PCT levels are transient, peaking around 48–72 hours before falling when compared with control patients with only minor injuries [5]. This is in sharp contrast to other inflammatory markers, such as CRP or ESR, which may remain elevated in trauma patients for more than 7 days [22].

A 2009 study from Castelli et al. demonstrated that the acute elevations in PCT at the time of injury blunt the diagnostic value of PCT as a marker for sepsis immediately following a traumatic injury. It should be noted, however, that patients with high PCT levels on admission were more likely to have septic complications* at some point* during their hospitalization, (5.4 ng/mL (2.9–25) versus 1.6 ng/mL (0.4–4.80) *P* < 0.001) [23]. A single center Chinese study from 2012 demonstrates similar results in pediatric patients [24].

Because the initial elevations in PCT levels are only transient monitoring for secondary elevations in PCT levels after 48 hours after initial injury has been explored as a potentially more accurate predictor of septic complications following traumatic injury. A 2012 single center study from Sakran et al. found that secondary elevations of PCT levels served as the only significant predictor of sepsis when compared to length of stay, ventilator days, or ISS (odds ratio 2.37 (1.23–4.61) *P* = 0.01) [25].

In review, PCT levels have several identifiable trends and predictive value in the setting of trauma. Consider the following.

- PCT levels rise acutely in response to the initial injury, secondary to a systemic inflammatory response, similar to that observed in burn patients.
- Initial elevations are transient, decreasing after 48 hours. During this period, higher serum PCT levels appear to indicate a poorer prognosis in patients.
- Secondary elevations in PCT levels during the post-trauma recovery period can be used as a significant marker for the onset of sepsis, suggesting a potential routine role for PCT in the management of trauma patients while obtained throughout a hospitalization.

While the current findings are promising, at this time, there is no concurrent evidence to support the same application in the pediatric trauma population.

## 5. Summary

The objective of this study was to explore the current evidence supporting the utilization of PCT as an adjunct tool for clinical decision making in pediatric patients in the setting of trauma and thermal injury, while reviewing the well-established role of PCT in the diagnosis of sepsis.

The utility of PCT levels as a prognostic indicator in traumatic injuries is incomplete in the pediatric population. Retrospective studies have identified a positive correlation between significantly elevated PCT levels and worse outcomes. There are not, however, identified cut-offs that could be used to direct additional intervention or therapy to improve outcomes, limiting the diagnostic value of PCT in these settings compared to validated models for predicting outcomes based on mechanism of injury. In future studies, with larger sample sizes, these initial elevations in PCT levels may have value as prognostic markers and could provide an objective measurement for injury severity.

The current value for PCT in pediatric trauma and burn patients appears to reside after the acute event. Kinetics studies demonstrate that serum procalcitonin levels rise rapidly and significantly in response to underlying inflammatory process and that the degree of elevation correlates directly with severity. In the setting of traumatic and thermal injuries, the initial insult is linked to rapid, transient elevations of PCT levels. Because the initial elevations are only transient, following serum levels throughout the hospitalization for secondary elevations may have a utility in detecting delayed onset of infections in these patients.

Compared to the adult populations, the body of literature supporting a role for PCT in pediatric trauma and burn patient is incomplete, leading to inappropriate generalizations that do not completely account for the different physiology observed in pediatric patients. Given the demonstrated value of PCT as an adjunct tool for clinical decisions making, it would be of great interest to see further studies targeted specifically to pediatrics, which targeted gaining a more complete picture of the fundamental behavior of procalcitonin levels, with the goal of identifying reproducible trends in PCT levels that could be used to offer an objective measure in the setting of traumatic injury.
